# Advancements in virtual reality for performance enhancement in combat sports: a mini-review and perspective

**DOI:** 10.3389/fpsyg.2025.1563212

**Published:** 2025-03-05

**Authors:** Yike Li, Hansen Li, Chun Jiang, Yuqin Su, Sijia Jiang, Guodong Zhang

**Affiliations:** ^1^Institute of Sport Science, College of Physical Education, Southwest University, Chongqing, China; ^2^School of Physical Education, Sichuan Agricultural University, Ya’an, China; ^3^Department of Police Tactics, Chongqing Police College, Chongqing, China; ^4^College of Physical Education, Chongqing University of Posts and Telecommunications, Chongqing, China; ^5^International College, Krirk University, Bangkok, Thailand

**Keywords:** virtual reality, combat sports, virtual training, reaction time, performance enhancement

## Abstract

This mini-review examines the role of Virtual Reality (VR) in enhancing athletes’ performance and reaction abilities in combat sports, aiming to highlight the advantages and potential benefits of VR technology for improving outcomes in various combat disciplines. We identified 13 relevant studies from the Web of Science and Scopus databases, encompassing disciplines such as fencing, taekwondo, karate, judo, and wrestling. The findings indicate that VR training can enhance athletes’ sports skills and physical fitness, correct improper movements, provide training feedback, and, in some cases, surpass the effectiveness of traditional training methods. It also significantly enhances athletes’ reaction capabilities. Even with these benefits, VR usage in combat training is still quite limited. Future research should focus on how to better leverage the advantages of VR technology in practical combat training for athletes, addressing the lack of tactile feedback, aiding athletes adapt to competition pressure caused by spectators, and examining whether there are gender differences in the use of this technology for training.

## Introduction

Virtual Reality (VR) refers to a computer-generated immersive environment based on head-mounted displays (HMDs) that allows users to experience a sense of “being there” (Bowman and McMahan, 2007), which has emerged as a promising tool for developing athletes’ decision-making abilities by enhancing their perceptual and cognitive skills, which in turn facilitates improved sports decision-making (Yunchao et al., 2023). Existing evidence suggests that, compared to traditional video training, VR training leads to more significant improvements in athletes’ decision-making performance and visual search behavior (Fortes et al., 2021; Pagé et al., 2019). Additionally, VR-based imagery training has proven to be more effective than conventional methods currently in use (Bedir and Erhan, 2021; Ross-Stewart et al., 2018). VR enables safe, repeatable training tasks with full control over the training environment, including stimuli and difficulty levels (Hoffmann et al., 2014). Another significant advantage of VR is its ability to offer personalized training experiences, allowing athletes to compete or train with others from around the world, regardless of their competitive level, gender, or age (Neumann et al., 2018). Currently, VR is mainly used in sports like ball games (Faure et al., 2020), target sports (e.g., dart throwing) (Mousavi et al., 2019), snow sports (Ko et al., 2020), and water sports (Farley et al., 2019), but it has received less attention in complex sports such as combat sports.

Combat sports are classified as open-skill sports (including boxing, karate, taekwondo, wrestling, martial arts, judo, Muay Thai, kickboxing, sanda, jiu-jitsu, fencing, Sambo, Aikido, and mixed martial arts), characterized by sudden environmental changes that require athletes to react in dynamic and unpredictable contexts (Russo and Ottoboni, 2019; Wang et al., 2013). In combat sports, reaction and decision-making abilities are critical to the outcome of matches (Gierczuk et al., 2017, Mori et al., 2002), and VR just can contribute to such training (Kittel et al., 2019; Petri et al., 2019a). Another key characteristic of combat sports is physical conflict (Vasconcelos et al., 2020), such as repeated head impacts (Di Virgilio et al., 2019; Lota et al., 2022), which can result in injuries (Lystad et al., 2021). Therefore, finding more scientific ways to ensure athletes’ safety and prolong their careers is crucial. In response to this need, VR is useful as it allows for safe and repeatable training tasks with high controllability (Miles et al., 2012).

Despite these explorations, the application of VR in combat sports remains an emerging field. Therefore, this mini-review is presented to underline the valuable findings in this regard and offer several recommendations based on our years of experience in teaching and training within this field.

## Methods

We used keyword searches in WOS and Scopus (covering titles, abstracts, and keywords). Our search query was: Topic = “virtual reality” or “VR” AND Topic = “Boxing” OR “boxer” OR “combat sport*” OR “karate” OR “taekwondo” OR “wrestling” OR “fencing” OR “martial art*” OR “judo” OR “jiu jitsu” OR “wushu” OR “kung fu” OR “Muay Thai” OR “Krav Maga” OR “Sambo” OR “Aikido” OR “kickbox*.”This search query was derived from a previous review on VR (Rojas-Sánchez et al., 2023) and previous reviews on combat sports (Chaabene et al., 2018; Vasconcelos et al., 2020; Worsey et al., 2019). We set the search period from the inception of the databases to April 21, 2024, with a language limit of English.

## Results

### Virtual reality implementation in different combat sports specialties

VR has been applied in various sports training programs (Richlan et al., 2023), including combat sports. Baek et al. (2003) were among the first to utilize VR technology in combat training, proposing a VR-based training framework comprising three components: motion-guided interfaces, posture-oriented motion redirection, and assessment and recommendation schemes for corrective feedback. Finally, a demonstration was conducted in fencing training, correcting the errors of holding the sword apart during fencing using redirection methods.

**Figure 1:**
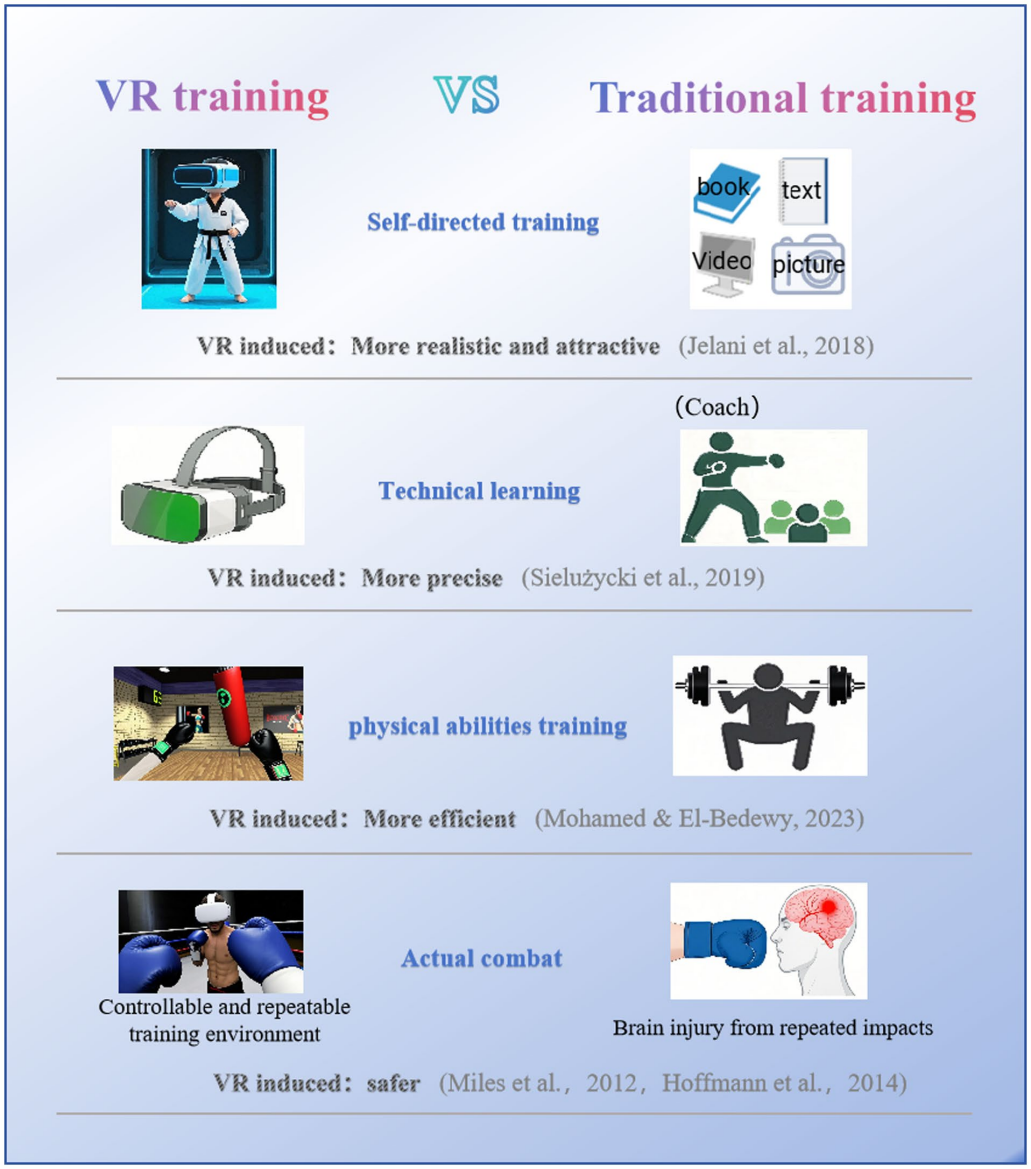
The comparison between virtual reality training and different traditional training methods.

In the field of Taekwondo, Jelani et al. (2018) developed a system to guide trainees in self-practice. The authors detailed four stages of this system, starting with motion capture of Taekwondo movements, followed by 3D modeling of characters and environments, animation of 3D characters, and finally, creating VR training environments. Compared to traditional Taekwondo training supplementary materials (books, videos, texts, images), VR training environments have significant advantages (see Figure 1). Another study indicated that VR technology improved the specific abilities of Taekwondo students in a short time frame, with significant improvements in high kicks and flying kicks. While VR technology assisted students in mastering challenging movements, it did not improve their physical fitness in a short time frame (standing long jump, sit-and-reach, 50-meter sprint, and 800-meter run) (Liu and Tian, 2024).

In Karate, researchers identified action sequences from 45 international Karate competitions, captured movements of three Karate athletes, and created the first practical Karate virtual training character (Zhang et al., 2018). This character can move in five specific directions and execute attacks close to reality based on the user’s actions. It passed functional and performance tests, and Karate experts believed it could improve athletes’ combat capabilities. Pastel et al. (2023) compared the effectiveness of visualizing different levels of body (full-body/forearm) visualization in Karate learners in VR environments versus video learning. The results showed significant improvements in all three groups after training, confirming VR as a suitable tool for learners to acquire sports techniques, but there were no significant differences among the three groups. Specifically, the VR group did not outperform the video group, and full-body visualization was not necessary as it did not show superiority over forearm visualization.

In Judo, researchers divided 24 Judo athletes into two groups and asked them to imitate three techniques of two world champions (masters) (Sielużycki et al., 2019). The experimental group underwent 5 VR (Kinect) training sessions, while the control group used Kinect for the first and fifth sessions, with the coach guiding the intermediate three sessions, each lasting 20 min. Kinect positioned 25 joints of the human body, scaled athletes relative to the masters’ height on the x, y, and z axes, and aligned them vertically to match the masters’ virtual joints on the screen. Athletes were prompted to simulate masters’ movements according to traffic lights and receive feedback to correct errors. The results showed significant improvement in the experimental group after five training sessions, outperforming the control group. Kinect significantly helped athletes’ movement accuracy, especially for elite athletes for whom slight differences could determine the outcome of a match. Most athletes had high expectations for the Kinect system’s entry into Judo clubs (Sielużycki et al., 2019).

In wrestling, Mohamed and El-Bedewy (2023) conducted an eight-week training program for novice wrestlers, three times a week, 90 min each, aiming to investigate the effectiveness of virtual reality training (VRT) in improving athletes’ physical abilities. The improvement in athletes’ back, leg, arm muscle strength, speed, flexibility, and agility after training was significantly better than that of the control group (traditional training group), with the greatest change in arm muscle strength and the least change in leg muscle strength. Researchers attributed the use of VR glasses in the training program as the main reason for this advantage and suggested incorporating VRT into wrestlers’ physical training to enhance training effectiveness.

In general, VR technology has been successfully integrated into various combat sports training programs, enhancing athletes’ skills through motion-guided interfaces, posture correction, and performance feedback, with evidence showing improvements in movement accuracy, technique mastery, and physical abilities, although its impact on overall fitness remains limited.

### The impact of virtual reality on reaction abilities in combat athletes

Regarding athletes’ reaction abilities, two studies conducted 10 sessions of virtual reality-specific reaction training for 15 Karate athletes, prompting them to respond to attacks from virtual opponents and testing reaction time, reaction quality, and reaction type (Petri et al., 2019a; Petri et al., 2019b). The conclusion was that VR training significantly improved Karate athletes’ reaction behaviors, such as shortening reaction time and transitioning from reacting to the execution phase of opponent attacks before training to reacting to early movement stages (shortening distance and preparation period) after training. The studies indicated that apart from lower reaction quality in virtual reality compared to the real world, there were no significant differences in reaction time and reaction type between the virtual and real worlds (Ritter et al., 2022). Adding 10 min of VR training to regular Karate training was beneficial for improving athletes’ reaction abilities, but it was not proven whether this benefit could transfer to the real world (Witte et al., 2022). Polechoński and Langer (2022) evaluated the reliability of using virtual reality technology to test mixed martial arts athletes’ reaction times, considering it equally applicable as computer-based standard tools, and effective in reducing testing errors caused by human factors (Langer et al., 2023). In general, VR training has been shown to significantly improve reaction times and reaction quality in Karate athletes, helping them react faster and more effectively to opponent movements, though the improvements may not directly transfer to real-world performance, and reaction quality in VR lags behind that in real-life scenarios.

## Discussion and future directions

VR technology has shown significant advantages in training. It can precisely record athletes’ movements and create virtual training avatars, allowing athletes to repeatedly practice and correct their techniques within an immersive virtual environment, thereby enhancing the accuracy and fluidity of their movements. At the same time, VR technology provides athletes with personalized training programs and feedback, enabling targeted training based on individual characteristics and needs, which strengthens the specificity and effectiveness of the training. Additionally, VR training has led to substantial improvements in athletes’ skills and physical fitness in a short period, such as enhancing high kick and flying kick abilities in taekwondo students, as well as increasing muscle strength and speed in wrestlers. In terms of reaction time, VR training has significantly improved athletes’ response behaviors, reducing reaction time and improving response quality. However, there is currently a lack of definitive evidence regarding the effective transfer of these advantages to real-world applications. A systematic review by Michalski et al. (2019) offers preliminary support for the effectiveness of VR training in enhancing real-world performance, which aligns with the findings reported by Harris et al. (2020). Despite these promising evidences, further validation of its impact on real-world performance is required.

A recent mini-review consolidates evidence that VR training facilitates patient rehabilitation, boosts energy expenditure and physical activity in individuals with disabilities (Li et al., 2025). Additionally, VR training has been demonstrated to effectively alleviate adolescent stress and enhance executive function (Cioffi et al., 2023). These results underscore VR’s extensive applicability.

Although VR has been shown to have significant advantages, there are also some unresolved limitations and different perspectives. Scientific information pertaining to the utilization of combat sports within Virtual Reality (VR) as an intervention for mentally impaired practitioners remains scarce. Potentially, this could represent a viable research trajectory for future endeavors. VR may assist individuals with mental disorders by enhancing their reaction times, attention, and decision-making. On the flip side, besides elite combat athletes, leisure and amateur practitioners deserve attention, comprising a large group. Many of them undertake training for fitness or leisure pursuits, rather than competitive endeavors. Studies have shown that the performance of the VR training group in throwing accuracy is significantly lower than that of the real-world training group, impairing real-world athletic performance (Drew et al., 2020). The efficacy of using VR technology to improve athletic skills is not high (Le Noury et al., 2022), indicating that not all sports are suitable for improving teaching or training effects through VR technology. For example, the lack of tactile feedback during use (Petri et al., 2019b) prevents athletes from launching second or even third attacks after blocking and defending like they do in the real world. The absence of tactile information may have a negative impact on the experience of combat sports users who require full-body contact (Berger et al., 2018), so adding tactile sensations in VR training is necessary for combat sports (Zhang et al., 2018). Only by giving athletes a more realistic sense of touch can they help transfer the training effects from virtual reality to the real world and competitions. Additionally, existing articles indicate that compared to traditional training, VR technology does not significantly improve physical fitness (Kong and Zhang, 2024; Pu and Yang, 2022; Wang et al., 2023). Interestingly, one study showed significant improvements in the physical abilities of wrestlers after 24 sessions of VR training (Mohamed and El-Bedewy, 2023). This may be related to the specific implementation details of VR intervention and differences in the devices used.

All training for combat athletes (such as physical fitness, reaction ability, and movement accuracy) ultimately serves to achieve superb combat capabilities. With the popularization of combat sports, the demands on athletes and coaches are increasing, and the demand for using emerging technologies to improve athletic skills is also growing. VR technology can provide significant advantages in real-world combat training because practicing in a virtual environment ensures athlete safety, eliminates their fear of injury, and allows them to challenge difficult techniques with greater confidence, which is crucial for athletes (especially high-level athletes) to improve their competitive abilities. Additionally, training against virtual opponents based on VR can reduce the psychological pressure of facing real opponents. Numerous athletes have ended their careers in training halls, and many athletes have suspended training after being injured in a single real-world combat. Some boxers tend to prove their superiority by using heavy hits, and opponents hit back with even greater force. In such real combat scenarios, they focus only on “surviving” rather than on better technique. At this point, they are in a state of mind known as “fight or flight,” which can create feelings of pressure, anxiety, and even panic. This psychological state greatly affects memory and learning, significantly impacting training effectiveness. That’s why some of the world’s great warriors, such as Tom Breese (Sherdog.com, 2024c), Donald Cerrone (Sherdog.com, 2024a), and Max Holloway (Sherdog.com, 2024b), no longer engage in real combat training. They found that using “games” (light sparring) as a substitute for real combat is more effective and safer. VR provides another safe and reliable training method for athletes. The increasing recognition of VR training as a performance enhancer in sports clubs is evident, exemplified by Chinese UFC athlete Wang Cong’s VR-based blurred vision simulation and Jake Paul’s use of VR during his preparation for a fight against Mike Tyson. These examples highlight the expanding use of VR in athletic training routines. It is crucial to highlight that VR cannot fully replace real-world social connections, teamwork, and adaptability. Ideally, VR should serve as a complementary tool rather than a complete substitute for in-person sports training.

Because of these advantages, we believe that VR-based virtual sparring may completely change the training process and improve athletes’ training experiences. Surprisingly, to our knowledge, only one paper (Zhang et al., 2018) directly discussed ways to use VR to improve the combat capabilities of karate athletes. Therefore, we suggest developing a virtual combat training system that creates opponents almost identical to real opponents, targeting different types of combat sports. For example, we can input videos of athletes’ matches or training sessions into the system, capturing opponents’ physique, technical styles, and tactical characteristics to build a database for creating virtual opponents. This will provide athletes with controlled opponents, a platform for repeatable practice, and safety assurance. They can autonomously switch between different virtual sparring partners, including world champion-level athletes they may not encounter in real life.

Secondly, by studying and analyzing videos of the world’s top athletes, we can construct a technical system to guide athletes in dealing with various opponents. Athletes can then engage in virtual combat training to truly master techniques and tactics, thereby breaking the limitations of traditional training thinking and resources. This also partially compensates for the shortage of coaches, as coaches in large sports teams often cannot provide one-on-one guidance to every trainee. What’s more, by simulating the cheers or boos of the audience, inexperienced athletes can better adapt to the pressure situations they may face during competitions. For instance, the presence or absence of spectators in a football stadium significantly impacts the psychological state, behavior, and performance of football players and teams (Richlan et al., 2023). Similar scenarios might also occur in combat sports arenas.

Finally, we found that most experimental subjects were male, with only a few studies including female participants. This aligns with the general under-representation of female participants in sports science (Anderson et al., 2023). Research by Kong and Zhang (2024) suggests that VR-assisted training is more effective for females than males, but it does not specifically explain the mechanisms and reasons behind this. To our knowledge, no other studies have focused on the gender differences in using this technology. Li et al. (2024) have pointed out that there is a severe gender imbalance in martial arts clubs, making it harder for female athletes to find suitable training partners. They often face challenges such as “excessive concessions” and concerns about “inappropriate physical contact” during training with male partners. Using VR training can effectively avoid these issues, which might be one of the reasons why training outcomes for females are better than for males. This also highlights the need for more attention to the application of these technologies to female groups in the future.

## Conclusion

We reviewed 13 studies on the use of VR in combat sports, summarizing its role in enhancing athletic performance. However, we believe that the broader advantages of this technology in combat sports training have yet to be fully realized. Accordingly, we concluded with several recommendations to encourage more researchers to explore the greater potential of VR in this field.
